# Disease-specific out-of-pocket and catastrophic health expenditure on hospitalization in India: Do Indian households face distress health financing?

**DOI:** 10.1371/journal.pone.0196106

**Published:** 2018-05-10

**Authors:** Anshul Kastor, Sanjay K. Mohanty

**Affiliations:** Department of Fertility Studies, International Institute for Population Sciences (IIPS), Deonar, Mumbai, India; National Institute of Technology Rourkela, INDIA

## Abstract

**Background:**

Rising non-communicable diseases (NCDs) coupled with increasing injuries have resulted in a significant increase in health spending in India. While out-of-pocket expenditure remains the major source of health care financing in India (two-thirds of the total health spending), the financial burden varies enormously across diseases and by the economic well-being of the households. Though prior studies have examined the variation in disease pattern, little is known about the financial risk to the families by type of diseases in India. In this context, the present study examines disease-specific out-of-pocket expenditure (OOPE), catastrophic health expenditure (CHE) and distress health financing.

**Methods and materials:**

Unit data from the 71^st^ round of the National Sample Survey Organization (2014) was used for this study. OOPE is defined as health spending on hospitalization net of reimbursement, and CHE is defined as household health spending exceeding 10% of household consumption expenditure. Distress health financing is defined as a situation when a household has to borrow money or sell their property/assets or when it gets contributions from friends/relatives to meet its health care expenses. OOPE was estimated for 16 selected diseases and across three broad categories- communicable diseases, NCDs and injuries. Multivariate logistic regression was used to understand the determinants of distress financing and CHE.

**Results:**

Mean OOPE on hospitalization was INR 19,210 and was the highest for cancer (INR 57,232) followed by heart diseases (INR 40,947). About 28% of the households incurred CHE and faced distress financing. Among all the diseases, cancer caused the highest CHE (79%) and distress financing (43%). More than one-third of the inpatients reported distressed financing for heart diseases, neurological disorders, genito urinary problems, musculoskeletal diseases, gastro-intestinal problems and injuries. The likelihood of incurring distress financing was 3.2 times higher for those hospitalized for cancer (OR 3.23; 95% CI: 2.62–3.99) and 2.6 times for tuberculosis patients (OR 2.61; 95% CI: 2.06–3.31). A large proportion of households who had reported distress financing also incurred CHE.

**Recommendations:**

Free treatment for cancer and heart diseases is recommended for the vulnerable sections of the society. Risk-pooling and social security mechanisms based on contributions from both households as well as the central and state governments can reduce the financial burden of diseases and avert households from distress health financing.

## Introduction

The demographic and epidemiological transition is universal irrespective of the level of socio-economic development within and between countries. As countries are converging to low levels of fertility and higher longevity, non-communicable diseases (NCDs) have become the leading cause of death in most of the countries. Globally, 40 million deaths are occurred due to NCDs each year (accounting for 70% of all deaths); this is projected to be 52 million by 2030 [1]. About half of the deaths due to NCDs were in low and middle-income countries, primarily affecting the working population (15–65 years) with dire social and economic consequences. It is estimated that NCDs and mental health conditions would cost US$ 30 trillion to the world economy between 2010 and 2030 if necessary steps would not be taken to prevent and treat them [2]. Reduction of NCDs is now a global and national priority and a prerequisite for attaining Sustainable Development Goals (SDGs) and reduction of poverty [3].

The changing disease pattern is associated with increasing hospitalisation and rising health care cost globally, nationally and locally. The economic consequences of rising NCDs for households and families are both direct (financial cost of health care) and indirect (loss of wage, income, and time of patient and caregiver) and vary by type and severity of illness [4–8]. In literature, two approaches are used to understand the economic hardship of health care payment- estimating catastrophic health spending and distress health financing [9–21]. In low and middle-income countries, out-of-pocket expenditure is the major source of financing health care and is catastrophic to a large number of households [13–17]. About 150 million people face financial catastrophe every year due to health care payments, out of which 90% reside in low-income countries [9,10]. The health financing system in low and middle-income countries is weak, causing a majority of the households to resort to selling assets, taking loans, borrowing money and getting contributions from friends and relatives to meet their health care expenses [18–23]

India is experiencing the triple burden of diseases, that is, rising non-communicable diseases, increasing injuries and the unfinished agenda of infectious diseases [2]. The disease pattern is changing rapidly, with non-communicable diseases (cardiovascular diseases, cancer, chronic respiratory diseases, diabetes, etc.) becoming the leading cause of mortality. NCDs in India accounted 50% of total deaths in 2004 and increased to 60% by 2014 [24,25]. Similarly, hospitalisation due to NCDs accounted for 29% of total hospitalisation in 2004 and increased to 38% by 2014. The share of out-of-pocket expenditure on total health spending has remained stagnant during the period (71% in 2004 and 69% in 2014) [26,27]. At the same time, the household health spending is growing faster than the household consumption expenditure [28]. An estimated 32–39 million people are pushed into poverty every year due to health care payments [11,29,30,31]. Many families face financial catastrophe and fall into poverty due to hospitalization costs [32].

The extent of disease-specific financial burden due to hospitalization is poorly researched in India. In recent years, a number of studies have estimated the out-of-pocket expenditure, CHE, determinants of CHE and impoverishment effect of OOPE [17,29,31,33]. However, most of these studies have used maternal health expenditure as the case. Studies have also documented that health spending is catastrophic to the poor, less educated, rural households, female-headed households and households with elderly members [34,35]. A number of studies have examined the extent of health expenditure on some specific diseases (diabetes, tuberculosis, cancer, injuries, etc.) but these provide the estimates only for small geographical areas with unrepresentative data [22,36–40] and, thus, cannot be generalised. Also, though there are some studies that estimated the financial burden of hospitalization for NCDs in India [8, 41], as per our knowledge, there is no study that has examined the disease-specific out-of-pocket- health expenditure on hospitalization taking into account both acute (fever, diarrhoea, etc.) and chronic diseases (cancer, heart diseases, diabetes, hypertension, etc.). We believe that the OOPE varies enormously by type of disease, health care provider (public/private), quality of care and geographical region in India. Treating cancer, heart disease, diabetes and injuries is not only expensive but also requires long hospitalisation and specialised treatment. With this backdrop, the present study estimates the out-of-pocket health expenditure on hospitalization by disease and its catastrophic impact on households in India. It addresses the research question as to what extent OOPE, CHE and distress health financing on hospitalization differ by disease and type of health care provider (public and private). Systematic estimation of OOPE, CHE and distress health financing by type of disease is helpful for evidence-based policy.

## Methods and materials

### Data and survey design

Unit data from social consumption as collected in the 71^st^ round of the National Sample Survey Organization (NSSO) was primarily used for this study. This is a nationally representative survey covered 333,104 individuals from 65,932 households (36,480 rural and 29,452 urban households) from January to June, 2014. A total of 42,869 hospitalization cases (excluding hospitalization due to childbirth) were reported and included in the analysis. Stratified multi-stage sampling design was used for data collection. Census villages in the rural areas and urban frame survey blocks in the urban areas were taken as the first stage units. Detailed information can be found in the survey report [42]. Information on expenditure on hospitalization (in a reference period of one year) we used included both medical and non-medical expenses. The medical expenses included health care provider’s fees, medicines, diagnostic tests (X-rays, ECG etc.), bed charges and other medical expenses (attendant charges, physiotherapy, personal medical appliances, blood, oxygen, etc.). The non-medical expenses included transport charges for the patient and for others, expenditure on food, expenditure on escort, lodging charges, etc. Hospitalization is defined as an overnight stay in the hospital anytime in 365 days prior to the survey. OOPE is defined as health expenditure net of reimbursement. The NSSO asked two subjective questions on the source of finance for expenses, and the sources being coded as household income/saving, borrowings, sale of physical assets, contribution from friends and relatives and other sources. Distress financing is defined as borrowing money, sale of property/assets or contributions from friends and relatives to cover the health care spending. The analyses were carried out by applying sampling weights provided by the NSSO.

### Disease classification

The NSS data provides the nature of ailments (diseases) into 60 categories (excluding childbirth). We reclassified these diseases into 16 broad categories based on the nature and type of the disease and their sample size (minimum 500 cases). The 16 categories included in the analysis are fever, gastro intestinal, injuries, genito urinary disorders, heart diseases, musculoskeletal, neurological, diarrhoea, asthma, cancer, cataract, hypertension, jaundice, respiratory disorders, diabetes and tuberculosis. These 60 categories were also grouped into three broad categories, namely, communicable/maternal/perinatal/nutritional diseases, non-communicable diseases and injuries as done in the report on the Causes of Death, India 2001–03 [43].The disease classification and coding are given in S1 Table.

### Outcome variables

The mean OOPE expenditure for each disease, the catastrophic health expenditure and distress health financing were used as outcome variables in the analyses. All three outcome variables were analysed for hospitalization only.

### Explanatory variables

The independent variables included in the analyses are age, sex, educational attainment, monthly per capita consumption expenditure (MPCE), educational attainment (no education, primary, secondary, higher secondary and above) place of residence (rural/urban) and type of health care provider (public/private). An MPCE tertile was derived and used in the analyses.

### Statistical methods

Descriptive statistics, estimation of CHE and logistic regression were used in the analyses. Mean, standard error and 95% confidence interval were provided for OOPE. Bivariate analyses were carried out to understand the differences in CHE by type of diseases. In literature, two alternative approaches are used in estimating catastrophic health spending, both using capacity to pay. The first approach, suggested by Berki (1986) and later by Van Doorslar et al. (2007), is a ratio method that defines CHE as a fraction of consumption expenditure (usually 10% and more) [11,12]. The second approach by Xu et al. (2003) defines CHE when a household’s health care spending exceeds 40% of its capacity to pay (non-subsistence consumption) [9]. The 71^st^ round, schedule 25 of NSS provides a single variable on consumption expenditure (it does not provide information on non-subsistence consumption); so we used the ratio method to define CHE. CHE is defined as *OOPE/HCE*> = 10—— (1), where *OOPE* is out-of-pocket health expenditure and *HCE* is household consumption expenditure. Logistic regression was used to understand the determinants of distress financing and catastrophic health expenditure.

## Results

### Socio-economic and demographic profile of the sample population

The socio-economic and demographic characteristics of the study samples are shown in Table 1. Out of the total sampled population- 48.5% (95% CI: 48.4–48.7) were females, 7.9% (95% CI: 28.8–29.1) were aged 60 and above and 30% (95% CI: 29.6–30.3) were residing in urban areas. With respect to educational attainment, 31.5% (95% CI: 31.4–31.7) did not have any formal education while 14.3% (95% CI: 14.1–14.4) had attained higher secondary+ education. The MPCE in urban areas (INR 2414; 95% CI: 2392–2436) was almost double than that in the rural areas (INR 1287; 95% CI: 1280–1295).

**Table 1:** Sampling profile of individuals and households in the 71^st^ round of National Sample Survey (2014), India.

Variable	2014	[95% CI]
**Age distribution**		
Child Population (0–14)	28.9	[28.8, 29.1]
Working Age (15–59)	63.2	[63.0, 63.3]
Elderly (60+)	7.9	[7.8, 8.0]
**Sex**		
Male	51.5	[51.3, 51.6]
Female	48.5	[48.4, 48.7]
**Place of Residence**		
Rural	70	[69.7, 70.4]
Urban	30.0	[29.6, 30.3]
**Education level**		
No Education	31.5	[31.4, 31.7]
Primary	30.3	[30.2, 30.5]
Secondary	23.9	[23.7, 24.0]
Higher Secondary	14.3	[14.1, 14.4]
**Mean MPCE (in INR)**^#^		
Combined (Rural + Urban)	1625	[1615, 1635]
MPCE (Rural)	1287	[1280, 1295]
MPCE (Urban)	2414	[2392, 2436]
Number of Households	65932	
Number of Persons	333104	
Number of Hospitalized Cases	42869	

^#^ 1 USD = 60.745 INR at 2014 exchange rates.

### Prevalence of disease-specific hospitalization rate in India

Table 2 presents hospitalization rate (per 100000 population) by type of disease in India. Overall, the hospitalization rate was 3700 per 100000 population, of which 62% was in private health care facilities and 38% in public health care facilities. For each of the diseases (except for tuberculosis), the hospitalization rate was higher in private health care facilities. The hospitalization rate was the highest for fever (659), followed by injuries (411) and gastro intestinal diseases (404). On the other hand, the lowest hospitalization was reported for tuberculosis (50), followed by jaundice (71) and diabetes (73). Heart disease, genito urinary and musculoskeletal diseases also showed significant hospitalization. Out of the total hospitalization cases in India, 38% were due to communicable diseases and 31% due to NCDs.

**Table 2:** Hospitalization rate per 100000 population (during 365 days prior to the survey) by diseases and health care provider (public/private) in India, 2014.

Diseases	Hospitalization Rate per 100000 Population		
	Public	Private	All
Diarrhea	68	56	124
Fever	246	413	659
Cataract	41	84	125
Tuberculosis	32	18	50
Respiratory	28	48	76
Asthma	53	62	114
Hypertension	42	68	110
Diabetes	23	50	73
Jaundice	29	43	71
Gastro intestinal	135	269	404
Neurological	61	91	153
Musculoskeletal	56	118	175
Genito urinary	62	187	249
Injuries	178	233	411
Heart Diseases	106	189	295
Cancer	35	52	87
**All Diseases**	**1422**	**2278**	**3700**
Communicable Diseases	607	806	1412
NCDs	386	755	1142
Injuries	178	233	411

### Duration of hospital stay

The average duration of hospital stay among the hospitalized was 7 days (SE: 0.052), with the figures being similar in public and private hospitals (7.2 days, SE: 0.091 and 6.9 days SE: 0.062 respectively) (S2 Table). The longest duration of stay was recorded for cancer inpatients (14.8 days, SE: 0.804) both in public (16.7 days, SE: 1.261) and private hospitals (13.6 days, SE: 1.043) followed by neurological problems and tuberculosis. Notably, the duration of hospital stay for neurological patients was twice as high in public hospitals as in private health centers. Cataract inpatients reported the lowest hospital stay (2.5 days, SE: 0.074) in both public (3.0 days, SE: 1.151) and private hospitals (2.2 days, SE: 0.079) compared to all other inpatients. Further, the mean duration of hospital stay was significantly higher for NCDs and injuries (8.6 days, SE: 0.121 and 8.9 days, SE: 0.202 respectively) than for communicable diseases (5.7 days, SE: 0.053).

### Disease-specific out-of-pocket expenditure on hospitalization

Tables 3 and 4 show mean total health spending, reimbursement and OOPE on hospitalization by diseases and health care provider (public/private). Average total spending, reimbursement and OOPE on hospitalization for all diseases were (INR 20370 (SE: 259), INR 1160 (SE: 103) and INR 19210 (SE: 237) respectively. On an average, only 5.7% (SE: 0.301) of the total spending on hospitalization was reimbursed (2.8%, SE: 0.123 in public hospitals and 6.2%, SE: 0.499 in private hospitals), with the highest reimbursement having been reported for genito urinary diseases (10.3%, SE: 3.610). The mean OOPE on hospitalization was 3.5 times higher in private health care facilities (INR 26407, SE: 370) than in public ones (INR 7583, SE: 191). The highest OOPE was reported for cancer (INR 57232, SE: 3885) both in public (INR 28281, SE: 2407) and private hospitals (INR 76375, SE: 6229), followed by heart diseases (INR 40947, SE: 1406) and injuries (INR 25003, SE: 758). The mean OOPE on hospitalization was noted to be INR 20000 or more for musculoskeletal, neurological, genito urinary and gastro intestinal diseases. The lowest OOPE was recorded for diarrhea (INR 5473, SE: 214), followed by fever (INR 8286, SE: 8286) and cataract (INR 9827, SE: 428). OOPE on NCDs (INR 28601, SE: 573) was about three times higher compared to that on communicable diseases (INR 10623, SE: 187).

**Table 3:** Mean spending on hospitalization, the amount reimbursed and out-of-pocket expenditure (in INR)^#^ and share of amount reimbursed to total health spending by disease in India, 2014.

Diseases	Total Spending on Hospitalization (Std. Err.)	Amount Reimbursed (Std. Err.)	OOPE (Std. Err.)	Reimbursed as a percentage of total health spending (Std. Err.)
Diarrhea	5640 (220)	167 (53)	5473 (214)	3.0 (0.207)
Fever	8708 (184)	422 (62)	8286 (171)	4.8 (0.896)
Cataract	10394 (436)	567 (104)	9827 (428)	5.5 (0.432)
Tuberculosis	13121 (888)	18 (24)	13104 (888)	0.1 (0.271)
Respiratory	14046 (755)	556 (181)	13490 (720)	4.0 (0.313)
Asthma	14851 (959)	1077 (232)	13774 (927)	7.3 (0.420)
Hypertension	14842 (1242)	536 (259)	14306 (1213)	3.6 (0.379)
Diabetes	15768 (790)	479 (145)	15290 (777)	3.0 (0.426)
Jaundice	18430 (1261)	636 (982)	17794 (1514)	3.5 (0.420)
Gastro intestinal	19633 (537)	1288 (143)	18345 (517)	6.6 (0.243)
Neurological	19616 (879)	969 (274)	18646 (822)	4.9 (0.300)
Musculoskeletal	24440 (1077)	1642 (374)	22798 (995)	6.7 (0.326)
Genito urinary	27150 (1476)	2807 (1248)	24343 (798)	10.3 (3.610)
Injuries	26361 (809)	1358 (208)	25003 (758)	5.1 (0.392)
Heart Diseases	43243 (1450)	2296 (352)	40947 (1406)	5.3 (0.471)
Cancer	62349 (4091)	5117 (1104)	57232 (3885)	8.2 (0.468)
**All Diseases**	**20370 (259)**	**1160 (103)**	**19210 (237)**	**5.7 (0.301)**
Communicable Diseases	11054 (188)	431 (67)	10623 (187)	3.9 (0.419)
NCDs	30661 (655)	2060 (309)	28601 (573)	6.7 (0.799)
Injuries	26361 (809)	1358 (208)	25003 (758)	5.1 (0.392)

^#^ 1 USD = 60.745 INR at 2014 exchange rates.

**Table 4:** Public-private differentials in mean spending on hospitalization, amount reimbursed and out-of-pocket expenditure (in INR) ^#^ and share of amount reimbursed to total health spending by disease in India, 2014.

Diseases	Total Spending		Amount Reimbursed		OOPE		Reimbursed as a percentage of total health spending (Std. Err.)	
	Public (Std. Err.)	Private (Std. Err.)	Public (Std. Err.)	Private (Std. Err.)	Public (Std. Err.)	Private (Std. Err.)	Public (Std. Err.)	Private (Std. Err.)
Diarrhea	2219 (98)	9766 (461)	13 (12)	354 (127)	2205 (94)	9412 (450)	0.6 (0.136)	3.6 (0.455)
Fever	3185 (174)	11964 (278)	43 (12)	648 (106)	3142 (173)	11316 (254)	1.3 (0.305)	5.4 (1.525)
Cataract	2203 (173)	14313 (603)	12 (15)	838 (159)	2191 (172)	13475 (599)	0.5 (0.204)	5.9 (0.646)
Tuberculosis	6692 (552)	24178 (1987)	14 (12)	24 (60)	6678 (552)	24154 (1987)	0.2 (0.421)	0.1 (0.172)
Respiratory	8422 (775)	17283 (1134)	259 (177)	727 (281)	8163 (728)	16555 (1085)	3.1 (0.310)	4.2 (0.483)
Asthma	5117 (302)	23200 (1714)	23 (13)	1981 (433)	5095 (302)	21218 (1666)	0.4 (0.141)	8.5 (0.766)
Hypertension	4175 (741)	21354 (1978)	53 (28)	832 (436)	4122 (741)	20523 (1932)	1.3 (0.566)	3.9 (0.509)
Diabetes	5797 (736)	20404 (1082)	254 (258)	584 (176)	5544 (667)	19820 (1074)	4.4 (0.447)	2.9 (0.601)
Jaundice	13120 (2574)	21958 (1094)	50 (23)	1030 (1706)	13070 (2575)	20928 (1819)	0.4 (0.483)	4.7 (0.637)
Gastro intestinal	6847 (478)	26046 (770)	398 (172)	1736 (201)	6449 (420)	24311 (751)	5.8 (0.309)	6.7 (0.337)
Neurological	9908 (618)	26119 (1407)	19 (18)	1609 (470)	9889 (618)	24510 (1310)	0.2 (0.115)	6.2 (0.506)
Musculoskeletal	10069 (1107)	31290 (1530)	328 (251)	2269 (569)	9741 (1056)	29021 (1407)	3.3 (0.221)	7.3 (0.492)
Genito urinary	11949 (863)	32202 (2017)	486 (110)	3581 (1741)	11463 (853)	28622 (1044)	4.1 (0.407)	11.1 (5.029)
Injuries	8928 (400)	39568 (1342)	240 (95)	2209 (361)	8689 (380)	37359 (1257)	2.7 (0.360)	5.6 (0.636)
Heart Diseases	15837 (1132)	58605 (2139)	826 (403)	3126 (506)	15011 (1063)	55479 (2083)	5.2 (1.143)	5.3 (0.326)
Cancer	29131 (2432)	84321 (6555)	850 (362)	7946 (1837)	28281 (2407)	76375 (6229)	2.9 (0.508)	9.4 (0.705)
**All Diseases**	**7802 (196)**	**28154 (408)**	**219 (40)**	**1747 (172)**	**7583 (191)**	**26407 (370)**	**2.8 (0.123)**	**6.2 (0.499)**
Communicable Diseases	4513 (185)	15927 (298)	58 (15)	711 (123)	4455 (184)	15216 (299)	1.3 (0.146)	4.5 (0.762)
NCDs	12756 (468)	39783 (975)	454 (123)	2881 (478)	12301 (450)	36902 (845)	3.6 (0.329)	7.2 (1.234)
Injuries	8928 (400)	39568 (1342)	240 (95)	2209 (361)	8689 (380)	37359 (1257)	2.7 (0.360)	5.6 (0.636)

^#^ 1 USD = 60.745 INR at 2014 exchange rates.

### Socio-economic and demographic differentials in disease-specific OOPE on hospitalization

Socio-economic and demographic differentials in mean OOPE on hospitalization by the disease are presented in S3 Table. The mean OOPE of the elderly was twice as high as that of the children. On an average, males spent 24% more on hospitalization than females. Individuals residing in urban areas reported higher spending (INR 24107, SE: 433) than those residing in rural areas (INR 16558, SE: 269). Education and MPCE tertile were positively associated with higher spending on hospitalization. The mean OOPE for those having a higher secondary+ education was at least twice as high as (INR 28449, SE: 857) compared to those with no formal education (INR13502, SE: 230). People in the richest tertile spent substantially higher amounts on hospitalization (INR30370, SE: 618) than the poor (INR 12391, SE: 234). The mean OOPE on hospitalization for all of the specified diseases was higher among males except for cataract and gastro intestinal problems. The OOPE on hypertension was almost three times higher among males than females. The OOPE on cancer was the highest followed, by heart diseases. The OOPE on cancer hospitalization was almost similar for urban and rural residents. The mean OOPE on heart diseases was three times higher among those having a higher education (INR 66323, SE: 4260) than among the less educated individuals (INR 21922, SE: 1347). Gastro intestinal, musculoskeletal, genito urinary and neurological diseases, as well as injuries, imposed a substantial financial burden irrespective of the level of education. Higher economic status was positively associated with OOPE on hospitalization for most of the diseases. Among all the diseases, lowest OOPE on hospitalization was reported for cataract among the poor.

### Disease-specific catastrophic health expenditure in India

**Fig 1:**
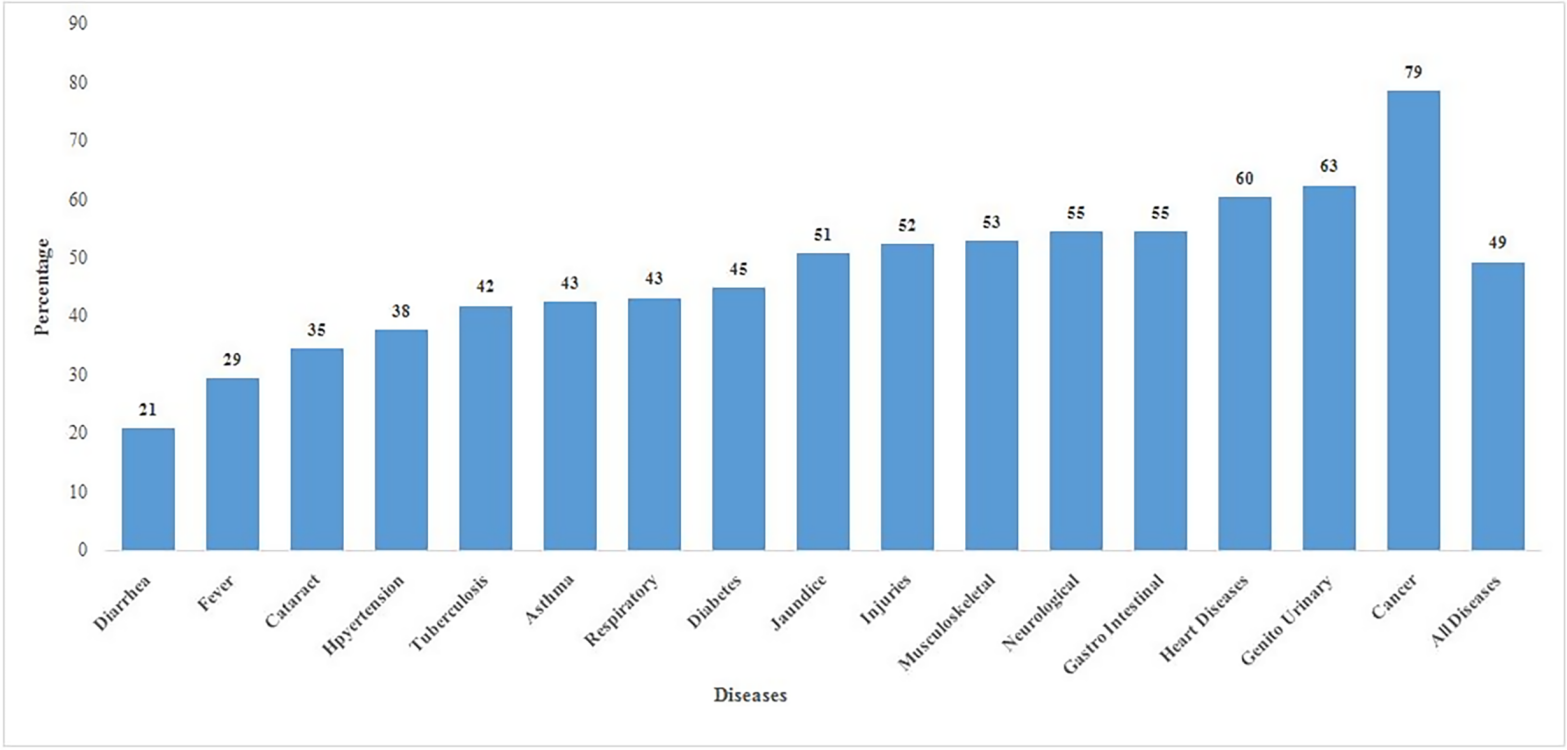
Percentage of households incurring catastrophic health expenditure on hospitalization by type of disease in India, 2014.

**Fig 2:**
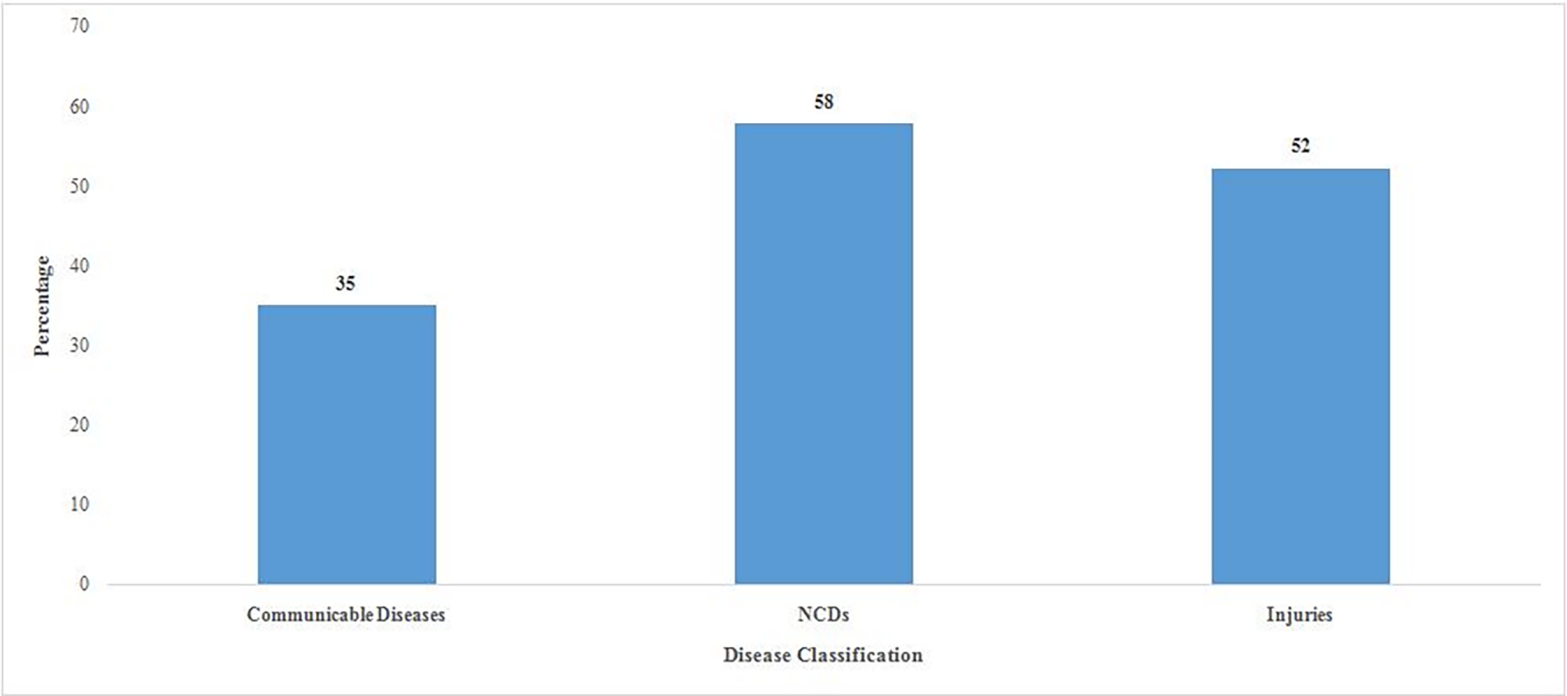
Percentage of households incurring catastrophic health expenditure on hospitalization by communicable diseases, NCDs and injuries in India, 2014.

Out of all the households who had any member hospitalized, 49% incurred catastrophic health expenditure (Fig 1). The highest incidence of CHE was reported for cancer (79%), followed by genito urinary (63%) and heart diseases (60%). The CHE was the lowest for diarrheal diseases (21%) and fever (29%) hospitalization. The CHE on hospitalisation was 58% for NCDs, 52% for injuries and 35% for communicable diseases (Fig 2).

Table 5 presents the results of logistics regression (odds ratio) on determinants of CHE on hospitalization. Households headed by individuals aged 60 years and above and female-headed households had higher chances of incurring CHE. The odds of incurring catastrophic health expenditure for any disease was significantly lower for urban residents (OR: 0.80; 95% CI: 0.76–0.83) than rural residents. The likelihood of CHE due to NCDs was 29% higher in households headed by an individual with higher secondary+ education (OR: 1.29; 95% CI: 1.13–1.46) than in households with an uneducated head. Further, odds of incurring CHE were significantly lower among the rich and middle MPCE households than among the poor.

**Table 5:** Odds ratio and 95% confidence interval for incurring catastrophic health expenditure on hospitalization for communicable diseases, NCDs, injuries and all diseases in India, 2014.

Background Characteristics	Communicable Diseases		NCDs		Injuries		All Diseases	
	Odds Ratio	95% CI	Odds Ratio	95% CI	Odds Ratio	95% CI	Odds Ratio	95% CI
**Age**								
<60 [Ref.]								
60+	1.17***	[1.07, 1.27]	1.07	[0.98, 1.16]	1.14	[0.98, 1.32]	1.25***	[1.19, 1.31]
**Sex**								
Male [Ref.]								
Female	1.05	[0.94, 0.18]	1.19***	[1.05, 1.34]	1.02	[0.82, 1.25]	1.09**	[1.02, 1.68]
**Place of Residence**								
Rural [Ref.]								
Urban	0.82***	[0.76, 0.89]	0.68***	[0.63, 0.74]	0.81***	[0.71, 0.93]	0.80***	[0.76, 0.83]
**Educational Level**								
No Education [Ref.]								
Primary	0.84***	[0.76, 0.93]	1.00	[0.90, 1.12]	0.85*	[0.72, 1.02]	0.91	[0.85, 0.96]
Secondary	0.92	[0.84, 1.02]	1.07	[0.96, 1.20]	0.90	[0.75, 1.06]	0.98	[0.92, 1.04]
Higher Secondary+	0.98	[0.87, 1.11]	1.29***	[1.13, 1.46]	0.88	[0.71, 1.08]	1.07*	[1.00, 1.15]
**MPCE Tertile**								
Poor [Ref.]								
Middle	0.82***	[0.75, 0.89]	0.84***	[0.75, 0.93]	0.77***	[0.66, 0.89]	0.89***	[0.84, 0.94]
Rich	0.75***	[0.68, 0.83]	0.84***	[0.75, 0.93]	0.78***	[0.66, 0.92]	0.90***	[0.85–0.96]

*p<0.1

**p<0.05 and

***p<0.01

Background Characteristics shown are of the household head.

### Disease-specific distress financing

Table 6 shows the percentage of hospitalization by disease and distressed financing. Out of all those hospitalized, 28.6% (95% CI: 28.2–29.0) reported distress financing and it was substantially higher among those who sought care in private hospitals (32.7%; 95% CI: 32.1–33.3) than in public hospitals (21.9%; 95% CI: 21.3–22.5). Distress financing was the highest for cancer hospitalization (42.5%; 95% CI: 39.7–45.4). Distress financing was the highest for cancer hospitalisation, both in private (47.8%; 95% CI: 44.1–51.6) and public health centres (34.5%; 95% CI: 30.3–38.8). Following cancer, distress financing was highest for tuberculosis in private health centres. Lowest distress financing was recorded for cataract both in public and private health centres. Similarly, one-third of hospitalization for NCDs resulted in distress financing.

**Table 6:** Distress financing (%) on hospitalization by diseases and health care provider (public-private) in India, 2014.

Diseases	Distressed Financing (%)		
	Public [95% CI]	Private [95% CI]	All [95% CI]
Diarrhea	14.6 [12.5, 16.7]	23.2 [20.0, 26.4]	18.5 [16.7, 20.3]
Fever	16.1 [14.9, 17.4]	26.9 [25.6, 28.3]	22.9 [22.0, 23.9]
Cataract	10.1 [07.1, 13.0]	18.4 [15.5, 21.3]	15.7 [13.5, 17.9]
Tuberculosis	24.0 [19.7, 28.3]	41.0 [34.6, 47.4]	30.2 [26.6, 33.9]
Respiratory	22.1 [18.0, 26.1]	33.1 [29.0, 37.2]	29.1 [26.1, 32.0]
Asthma	19.1 [16.0, 22.3]	27.0 [23.7, 30.4]	23.4 [21.1, 25.7]
Hypertension	9.7 [06.9, 12.5]	25.1 [21.5, 28.6]	19.2 [16.8, 21.7]
Diabetes	17.4 [13.2, 21.5]	34.7 [30.8, 38.6]	29.2 [26.2, 32.2]
Jaundice	25.8 [21.6, 30.1]	31.8 [27.8, 35.8]	29.4 [26.5, 32.3]
Gastro intestinal	24.4 [22.4, 26.3]	38.9 [37.1, 40.6]	34.0 [32.7, 35.3]
Neurological	32.7 [29.4, 35.9]	36.0 [33.0, 38.9]	34.6 [32.4, 36.8]
Musculoskeletal	26.9 [23.8, 30.0]	32.8 [30.1, 35.4]	30.9 [28.8, 32.9]
Genito urinary	24.2 [21.4, 27.0]	38.0 [35.8, 40.1]	34.6 [32.8, 36.3]
Injuries	28.9 [26.9, 30.8]	35.6 [33.8, 37.5]	32.7 [31.4, 34.1]
Heart Diseases	27.2 [24.9, 29.6]	40.0 [37.9, 42.0]	35.4 [33.8, 37.0]
Cancer	34.5 [30.3, 38.8]	47.8 [44.1, 51.6]	42.5 [39.7, 45.4]
**All Diseases**	**21.9 [21.3, 22.5]**	**32.7 [32.1, 33.3]**	**28.6 [28.2, 29.0]**
Communicable Diseases	17.4 [16.6, 18.2]	28.1 [27.1, 29.0]	23.5 [22.9, 24.2]
NCDs	25.7 [24.5, 26.9]	36.7 [35.7, 37.8]	33 [32.2, 33.8]
Injuries	28.9 [26.9, 30.8]	35.6 [33.8, 37.5]	32.7 [31.4, 34.1]

Table 7 presents the unadjusted and adjusted odds ratios of distress financing on hospitalization. Noticeably, the odds ratio of all of the selected diseases did not show any significant variation even after adjusting for socio-economic and demographic covariates Compared to diarrhea, all of the selected diseases showed higher odds of distress financing. The likelihood of incurring distress financing was 3.2 times higher for those hospitalized for cancer (OR 3.23; 95% CI: 2.62–3.99) and 2.6 times for tuberculosis patients (OR 2.61; 95% CI: 2.06–3.31) than the diarrheal inpatients. Similarly, the likelihood of incurring distress financing for treating heart diseases was more than twice as compared to diarrheal diseases (OR: 2.43; CI: 2.06–2.87). The chances of distress financing were significantly higher for those who were hospitalized for NCDs and injuries (OR 1.55; 95% CI: 1.47–1.63 and OR 1.65; 95% CI: 1.52–1.79 respectively) than those hospitalized for communicable diseases (not shown in the table). The odds of distress financing declined with each gradient of educational level and MPCE tertile. The likelihood of distress financing was 2.3 times higher among inpatients admitted in private hospitals (OR 2.30; 95% CI: 2.19–2.43) than those in public hospitals.

**Table 7:** Unadjusted odds ratio, adjusted odds ratio and 95% confidence interval of incurring distress financing on hospitalization for specific diseases in India, 2014.

Covariates	Unadjusted Odds Ratio	95% CI	Adjusted Odds Ratio	95% CI
**Diseases** Diarrhea [Ref.]	** **		** **	
Fever	1.35***	[1.17, 1.57]	1.29***	[1.11, 1.50]
Gastro intestinal	2.03***	[1.75, 2.36]	1.88***	[1.61, 2.20]
Injuries	2.47***	[2.13, 2.87]	2.38***	[2.03, 2.78]
Genito urinary	2.46***	[2.10, 2.87]	2.23***	[1.89, 2.63]
Heart Diseases	2.42***	[2.07, 2.82]	2.43***	[2.06, 2.87]
Musculoskeletal	1.99***	[1.68, 2.36]	1.87***	[1.56, 2.23]
Neurological	2.56***	[2.17, 3.04]	2.37***	[1.99, 2.83]
Asthma	1.79***	[1.48, 2.16]	1.65***	[1.35, 2.02]
Cancer	3.18***	[2.66, 3.82]	3.23***	[2.62, 3.99]
Cataract	1.22*	[0.99, 1.50]	1.09	[0.88, 1.36]
Hypertension	1.50***	[1.22, 1.84]	1.58***	[1.27, 1.96]
Jaundice	2.07***	[1.70, 2.53]	1.82***	[1.47, 2.25]
Respiratory	1.76***	[1.43, 2.17]	1.65***	[1.33, 2.05]
Diabetes	2.06***	[1.69, 2.52]	2.05***	[1.65, 2.54]
Tuberculosis	2.67***	[2.14, 3.33]	2.61***	[2.06, 3.31]
Others	1.80***	[1.56, 2.10]	1.76***	[1.50, 2.05]
**Age**				
0–14 [Ref.]				
15–59			1.32***	[1.23, 1.42]
60+			0.99	[0.91, 1.08]
**Sex**				
Male [Ref.]				
Female			0.79***	[0.75, 0.83]
**Educational Level**				
No Education [Ref.]				
Primary			0.84***	[0.79, 0.89]
Secondary			0.63***	[0.59, 0.68]
Higher Secondary+			0.39***	[0.36, 0.43]
**MPCE Tertile**				
Poor [Ref.]				
Middle			0.81***	[0.77, 0.86]
Rich			0.66***	[0.62, 0.70]
**Place of Residence**				
Rural [Ref.]				
Urban			0.76***	[0.72, 0.80]
**Health Care Provider**				
Public [Ref.]				
Private			2.30***	[2.19, 2.43]

*p<0.1 and

***p<0.01

### Consistency of CHE and distress financing

**Fig 3:**
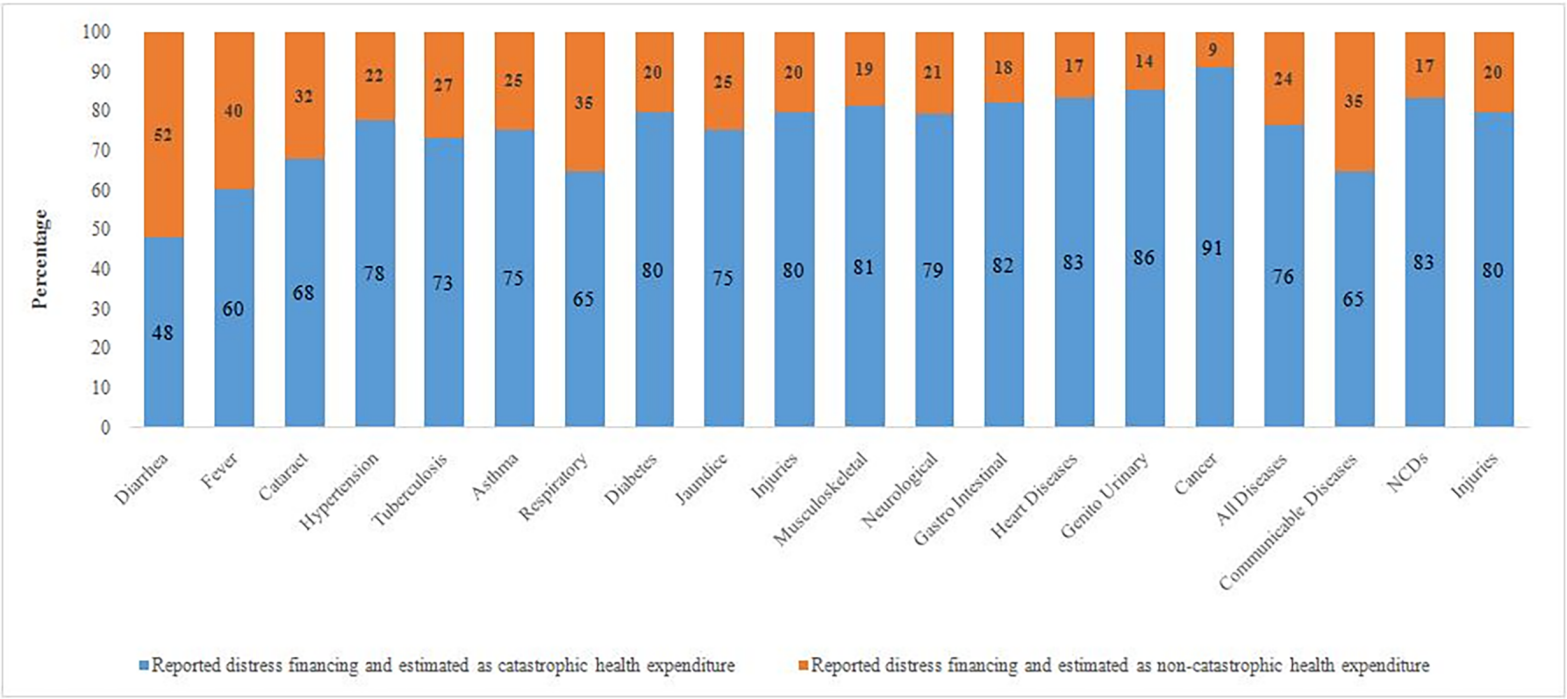
Percent distribution of distress financing by catastrophic and non-catastrophic health spending and type of disease in India, 2014.

Fig 3 presents the cross-tabulation of distress financing and CHE by type of disease. Since CHE is a numerical estimate and distress financing is a subjective measure, we cross-classified the extent of CHE by distress financing. Among all those who resorted to distress financing, 76% had CHE, which was highest for cancer (91%), followed by genito urinary and heart diseases. This shows that both distress financing and CHE are helpful to derive valid inferences on financial hardship of households in India.

## Discussion and conclusion

Rising burden of NCDs, increasing hospitalisation rate and health care cost and household health spending, warrants disaggregated analyses of financial catastrophe by type of disease and nature of health care providers in India. This paper is a first-ever attempt at providing disaggregated estimates of OOPE and CHE on hospitalization by type of disease in public and private health centres and at integrating the estimates of OOPE and CHE with distress financing. We have used the latest nationally representative survey data and excluded child birth related hospitalisation from the analyses. The following are our salient findings.

First, the total hospitalisation rate was 3700 (per 100,000 population), of which 1412 was for communicable diseases, 1142 for NCDs and 411 for injuries. A higher proportion of the population was hospitalized in private health centres (2278) compared to public health centers (1422), suggesting that private health centers are the main service providers for hospitalisation in India. Hospitalization was found to vary by type of disease and type of health centre. The hospitalisation for tuberculosis was higher in public health centers than private health centers, whereas for heart diseases, it was higher in private health centers.

Second, the mean cost of treatment, reimbursement and OOPE were found to vary largely by disease and type of health service providers. The average spending on hospitalization, the amount reimbursed and OOPE was highest for cancer, followed by heart diseases and injuries. The OOPE on hospitalization was higher among males than females. It was higher in the richest quintile than in the poorest quintile and among the better-educated individuals, establishing a link between the ability to pay and the quality of health care services. The OOPE in private health centers was at least thrice as high as in public health centers for most of the diseases, and the difference was the largest for cataract, followed by hypertension (in relative terms). Reimbursement was found to account for a very small share of household health spending in India.

Third, the extent of catastrophic health spending was found to vary largely across diseases and by type of health care provider. The catastrophic impact was the lowest for diarrheal diseases (21%), followed by fever (29%) and the highest for cancer (79%), followed by genital urinary (63%) and heart diseases (60%).

Fourth, more than one-third of the inpatients reported distress financing for being hospitalized for neurological disorders, genito urinary problems, musculoskeletal diseases, gastro-intestinal problems and injuries. The extent of distress financing was relatively higher among those who sought care from private health centres. Among all diseases treated in private health centres, the extent of distress financing was the highest for cancer (48%) followed by heart diseases (40%). Similarly, among those who sought care in public health centers, the extent of distress financing was the highest for cancer, followed by injuries.

Fifth, we found a great deal of agreement between distress financing and catastrophic health expenditure. While CHE is based on numerical estimation, distress financing is based on qualitative information. Among those who reported distress financing, more than three-fourths incurred catastrophic health expenditure. This was the highest for cancer and lowest for diarrheal diseases. In the case of NCDs, 85% of those who reported distress financing also incurred CHE, while this proportion was lower for communicable diseases.

We put forward some limitations of this study. The analyses were limited to inpatient care only (hospitalization). This may have resulted in an underestimation of the OOPE, CHE and distressed health financing of households. The data provides inpatient (hospitalization) expenditure for 365 days prior to the survey and outpatient expenditure for 15 days only. We know that health is an uncertain event so it is not feasible to adjust any of the two expenditures in either way to make it uniform (15 or 365 days). Secondly, recall bias may be higher in the 365 days reference period. Third, we have measured the CHE based on household health spending exceeding 10% of consumption expenditure. This method is not free from limitations as the method is not sensible to the health spending of the poor [29]. Moreover, any small spending on health for those living below the poverty line (say less than 10%) is catastrophic. Also, the threshold of the catastrophic health expenditure is unavoidably arbitrary and only a normative choice [44]. The health survey data of NSSO does not collect detailed consumption expenditure; therefore applying WHO method is not feasible. Further, the data does not provide any information on the amount of borrowings/debts, the cost of borrowings (interest rates) and their mode of repayment and, hence, the robust estimates of distress health financing could not be quantified. Despite these limitations, we believe that the paper contributes to the literature by providing disease-specific estimates from public and private health providers and establishing consistency of distress financing and CHE.

Our findings are consistent with those of the earlier studies. Higher hospitalization for NCDs in private health centres has also been found in other studies. Irrespective of the economic status, people prefer to avail health care services from private health centres [8], primarily due to the poor quality of care in public hospitals [39,40]. A substantial proportion of indebtedness in India is attributable to people’s preference for private hospitals [31,45]. Though the diagnosis and treatment of tuberculosis are free of charge in public health centres through the Revised National Tuberculosis Control Program (RNTCP) run by the Government of India [46], a quarter of those hospitalised for the disease reported to have resorted to distress financing. This may be because tuberculosis is more prevalent among the poorer sections [47] and even a small proportion of health expenditure for them would be financed through borrowings or selling the property. Our findings of higher OOPE among the richer and the better-educated people link health spending to the ability to pay and quality of care. It has been documented in the previous studies that the OOPE is much higher among the richer sections not only in India but also in developed countries like USA and Canada [28,48]. However, the impoverishment effect is least evitable among them [35]. The highest distress financing in our study was noted for cancer inpatients, followed by heart diseases, while the lowest was recorded for cataract and diarrheal inpatients. Joe (2015) has also concluded that households in India are at greater risk of indebtedness while seeking treatment for cancer and CVDs [23]. The long duration of inpatient stay coupled with a high treatment cost of these diseases not only increases the hospitalization charges but also aggravates other indirect expenses on food, lodging and transportation of escorts, which pushes up the total OOPE.

In the last one decade, there have been systematic efforts by the Government of India to improve the health services provided to the people. In 2005, the Government of India launched the National Rural Health Mission, now renamed as National Health Mission (NHM), to reduce maternal, neonatal and infant mortality. The NHM has been successful in increasing the utilisation and quality of maternal and child health care services and reducing the maternal and infant mortality rate [49,50]. Provision of care for NCDs, however, has remained untouched from the main agenda of the NHM. In 2008, the Government of India launched the *Rashtriya Bima Swasthya Yojana* (RBSY), a national health insurance scheme for the Indian poor. The main aim of the RSBY is to provide health insurance coverage to the families (maximum up to five members) who are below the poverty line (BPL), and to provide them access to quality health care and to protect them from catastrophic health expenditure. The scheme aims to enhance poor people’s choice of health care provider by empanelling both public and private hospitals. It provides cashless insurance of up to INR 30000 (US$450) per family per year for hospitalization in any of the empanelled hospitals [51]. However, macro studies have documented that the RSBY has not been successful in reducing the OOPE and catastrophic impact on the families [52]. The central government has also introduced some other social health insurance schemes such as the *Aam Aadmi Bima Yojana* (social security scheme for rural landless households) and the Universal Health Insurance Scheme (for poor families). The Central Government Health Scheme (CGHS) provides health care facilities to the central government employees and the pensioners and their dependents [53]. A number of schemes have also been launched by some states in India to provide health insurance primarily to poor families. For example, the *Rajiv Aarogyasri* Scheme in Andhra Pradesh provides financial protection to the families living below the poverty line up to US$ 3292 a year for the treatment of serious ailments requiring hospitalization and surgery. Likewise, the Gujarat government has launched the *Mukhyamantri Amrutam* scheme which provides quality medical and surgical care to the below poverty line families for catastrophic illnesses involving hospitalization, surgeries and therapies through an impanelled network of hospitals. The Chief Minister's Comprehensive Health Insurance Scheme in Tamil Nadu provides free medical and surgical treatment (up to US$ 2469 per family per year) in government and private hospitals to the members of families with an annual income less than US$ 1185 [54]. It needs to be mentioned that less than 20% of the population is covered under any health insurance scheme in India [55], and many of the health insurance schemes do not cover chronic illnesses [56] and, hence, may not reduce the OOPE and CHE in certain households. The Ministry of Health and Family Welfare, Government of India launched the National Programme for Prevention and Control of Cancer, Diabetes, Cardiovascular Disease and Stroke (NPCDCS) in 2010. Initially, the programme was implemented in only 100 districts covering 21 states. It was later expanded to many other districts across the country with focus on strengthening of infrastructure, human resource development, health promotion, early diagnosis, treatment and referral for prevention and control of cancer, diabetes, cardiovascular diseases and stroke [57]. The recently released National Health Policy, 2017 aims to increase the central government spending on health form the current level of 1.15% to 2.5% of GDP by 2025. The policy envisages to attain the highest possible level of health and well-being for all at all ages and to provide affordable and universal access to good quality health care services without anyone facing financial catastrophe. It specifically states that it aims to reduce the proportion of households incurring catastrophic health expenditure from the current level by 25%, by the year 2025 [58]. However, the success of the policy depends on how well it would be implemented across the country which has always been a big hurdle in Indian context.

### Conclusion

Disaggregation of OOPE and CHE combined with distress financing by disease and type of provider calls for financial protection in national and state health policies. We suggest for including treatment of cancer, heart diseases, and other rare and incurable diseases in the ambit of the health insurance coverage. It is also suggested to provide free treatment to the vulnerable segment of the population for the treatment of cancer and heart diseases, which was already done for treatment of tuberculosis in India. Diseases like neurological problems, musculoskeletal disorders and genito-urinary diseases also need special attention when framing policies as these diseases also impose a huge financial burden on the families. The coverage and the insurance amount of the RSBY are very low and need to be enlarged. The expansion of the NPCDCS to all the districts may be helpful in preventing many households from falling into the medical poverty trap.

## Supporting information

S1 Table Disease classification and coding of the NSS data used in the analysis. (DOCX)

S2 Table Mean duration of hospital stay (days) by diseases and health care provider (public-private) in India, 2014. (DOCX)

S3 Table Socio-economic and demographic differentials in mean OOPE on hospitalization (in INR)^#^ by diseases in India, 2014. (DOCX)

S1 Dataset NSS 71_working_hospitalization file.dta. (DTA)
