# Supplementary material for: Disease-specific out-of-pocket and catastrophic health expenditure on hospitalization in India: Do Indian households face distress health financing?
Source: PLoS One. 2018 May 10;13(5):e0196106. doi: 10.1371/journal.pone.0196106 (PMC5945043; doi:10.1371/journal.pone.0196106)
Supplement: S1 Table — (DOCX) [file pone.0196106.s001.docx]

**S1 Table. Disease classification and coding of the NSS data used in the analysis.**

| **Disease Classification** | **Disease Coding** |
| --- | --- |
| Communicable/ Maternal/ Perinatal / Nutritional Conditions | Fever with loss of consciousness or altered consciousness; Fever with rash/ eruptive lesions; Fever due to diphtheria; whooping cough; All other fevers (Includes malaria, typhoid and fevers of unknown origin, all specific fevers that do not have a confirmed diagnosis); Tuberculosis; Filariasis; Tetanus; HIV/AIDS; Other sexually transmitted diseases; Jaundice; Diarrhea dysentery/ increased frequency of stools with or without blood and mucus in stools; Worms infestation; Anaemia (any cause); Bleeding disorders; Under-nutrition; Goitre and other diseases of the thyroid; Acute upper respiratory infections (cold, runny nose, sore throat with cough, allergic colds included); Cough with sputum with or without fever and not diagnosed as TB; Pregnancy with complications before or during labour (abortion, ectopic pregnancy, abortion, hypertension, complications during labour); Complications in mother after birth of child; Illness in the newborn/ sick newborn. |
| Non-Communicable Diseases | Cancer;, Diabetes; Psychiatric & Neurological (mental retardation/ mental disorders/ seizures or known epilepsy/ weakness in limb muscles and difficulty in movements/ hemiplegia/ sudden onset weakness or loss of speech in half of body/memory loss etc.); Hypertension; Heart disease; Asthma; Musculo-skeletal (joint or bone disease/ pain or swelling in any of the joints, or swelling or pus from the bones); Genito-urinary (Any difficulty or abnormality in urination/ Pain the pelvic region/reproductive tract infection/ Pain in male genital area/ irregularity in menstrual cycle or excessive bleeding/pain during menstruation and any other gynaecological and andrological disorders incl. male/female infertility). |
| Injuries | Accidental injury, road traffic accidents and falls; Accidental drowning and submersion; Burns and corrosions; Poisoning; Intentional self-harm; Assault; Contact with venomous/harm-causing animals and plants. |
