# Supplementary material for: Disease-specific out-of-pocket and catastrophic health expenditure on hospitalization in India: Do Indian households face distress health financing?
Source: PLoS One. 2018 May 10;13(5):e0196106. doi: 10.1371/journal.pone.0196106 (PMC5945043; doi:10.1371/journal.pone.0196106)
Supplement: S2 Table — (DOCX) [file pone.0196106.s002.docx]

**S2 Table. Mean duration of hospital stay (days) by diseases and health care provider (public-private) in India, 2014.**

| **Diseases** | **Mean duration of hospitalization (Days)** | | |
| --- | --- | --- | --- |
|  | **Public (Std. Err.)** | **Private (Std. Err.)** | **All (Std. Err.)** |
| Diarrhea | 3.8 (0.086) | 4.1 (0.131) | 3.9 (0.074) |
| Fever | 5.2 (0.078) | 5.0 (0.069) | 5.1 (0.052) |
| Cataract | 3.0 (0.151) | 2.2 (0.079) | 2.5 (0.074) |
| Tuberculosis | 11.1 (0.895) | 9.0 (0.517) | 10.3 (0.594) |
| Respiratory | 6.2 (0.292) | 5.7 (0.242) | 5.9 (0.186) |
| Asthma | 8.1 (0.343) | 6.9 (0.261) | 7.4 (0.213) |
| Hypertension | 4.9 (0.204) | 4.7 (0.171) | 4.8 (0.179) |
| Diabetes | 8.0 (0.405) | 7.5 (0.501) | 7.7 (0.357) |
| Jaundice | 9.1 (0.546) | 9.5 (0.442) | 9.3 (0.344) |
| Gastro Intestinal | 6.5 (0.204) | 6.9 (0.131) | 6.7 (0.111) |
| Neurological | 14.9 (1.104) | 7.3 (0.322) | 10.4 (0.506) |
| Musculoskeletal | 9.2 (0.372) | 7.8 (0.251) | 8.2 (0.209) |
| Genito Urinary | 8.5 (0.306) | 7.7 (0.215) | 7.9 (0.176) |
| Injuries | 8.1 (0.279) | 9.5 (0.285) | 8.9 (0.202) |
| Heart Diseases | 8.3 (0.313) | 8.0 (0.194) | 8.1 (0.169) |
| Cancer | 16.7 (1.261) | 13.6 (1.043) | 14.8 (0.804) |
| **All Diseases** | **7.2 (0.091)** | **6.9 (0.062)** | **7.0 (0.052)** |
| Communicable Diseases | 5.7 (0.080) | 5.7 (0.071) | 5.7 (0.053) |
| NCDs | 9.9 (0.247) | 7.9 (0.128) | 8.6 (0.121) |
| Injuries | 8.1 (0.279) | 9.5 (0.285) | 8.9 (0.202) |
