# Supplementary material for: Disease-specific out-of-pocket and catastrophic health expenditure on hospitalization in India: Do Indian households face distress health financing?
Source: PLoS One. 2018 May 10;13(5):e0196106. doi: 10.1371/journal.pone.0196106 (PMC5945043; doi:10.1371/journal.pone.0196106)
Supplement: S3 Table — (DOCX) [file pone.0196106.s003.docx]

**S3 Table. Socio-economic and demographic differentials in mean OOPE on hospitalization (in INR)** ^#^ **by diseases in India, 2014.**

| **Diseases** | **OOPE on Hospitalization** | | | | | | | | | | | | | |
| --- | --- | --- | --- | --- | --- | --- | --- | --- | --- | --- | --- | --- | --- | --- |
|  | **Age-Group** | | | **Sex** | | **Education Level** | | | | **MPCE Tertile** | | | **Place of Residence** | |
|  | **0-14 (Std. Err.)** | **15-59 (Std. Err.)** | **60+ (Std. Err.)** | **Male (Std. Err.)** | **Female (Std. Err.)** | **No Education (Std. Err.)** | **Primary (Std. Err.)** | **Secondary (Std. Err.)** | **Higher Secondary (Std. Err.)** | **Poor (Std. Err.)** | **Middle (Std. Err.)** | **Rich (Std. Err.)** | **Rural (Std. Err.)** | **Urban (Std. Err.)** |
| Diarrhea | 5113 (242) | 5922 (395) | 5193 (601) | 5840 (282) | 5000 (295) | 4715 (220) | 4154 (273) | 5212 (523) | 13236 (1411) | 5805 (360) | 4445 (239) | 6648 (578) | 4471 (227) | 7295 (409) |
| Fever | 7979 (278) | 8735 (244) | 6918 (413) | 8708 (257) | 7367 (192) | 6925 (196) | 7327 (309) | 8900 (348) | 11619 (646) | 6815 (287) | 8173 (230) | 10246 (380) | 7857 (219) | 9109 (275) |
| Cataract | 64598 (8487) | 7614 (429) | 8851 (444) | 7074 (427) | 11670 (677) | 9639 (671) | 8823 (736) | 9420 (728) | 14988 (1170) | 4208 (359) | 5823 (436) | 18514 (962) | 6783 (393) | 16229 (857) |
| Tuberculosis | 12904 (2002) | 13815 (1186) | 10857 (1410) | 13615 (1316) | 11259 (1318) | 9940 (1304) | 12619 (1708) | 18764 (2501) | 17364 (4089) | 12304 (1182) | 9407 (1053) | 21387 (2788) | 11451 (983) | 17181 (1832) |
| Respiratory | 11003 (868) | 14788 (983) | 15353 (2306) | 13249 (992) | 13150 (906) | 11014 (919) | 11321 (1382) | 15824 (1409) | 22190 (2924) | 9996 (1011) | 11721 (924) | 19941 (1774) | 12136 (720) | 16387 (1510) |
| Asthma | 8429 (1054) | 11666 (762) | 16720 (1737) | 15415 (1630) | 11553 (969) | 9246 (654) | 18127 (3004) | 11446 (1005) | 29102 (4807) | 8650 (641) | 9060 (595) | 23396 (2572) | 13217 (1329) | 14721 (1258) |
| Hypertension | 15165 (10468) | 14311 (1519) | 14298 (1945) | 21242 (2192) | 7832 (613) | 10395 (1790) | 15678 (2254) | 19335 (2678) | 11007 (1917) | 9665 (2075) | 12255 (1390) | 20079 (2444) | 14132 (1943) | 14560 (1520) |
| Diabetes | 10641 (1186) | 14480 (1122) | 16300 (1125) | 16796 (1273) | 12532 (896) | 11952 (1102) | 16171 (1675) | 13505 (1279) | 18603 (2446) | 9413 (697) | 13430 (1133) | 18756 (1409) | 14082 (1064) | 16571 (1128) |
| Jaundice | 11188 (1165) | 21236 (2586) | 21562 (2490) | 20025 (2352) | 13395 (1090) | 12629 (1069) | 19823 (1424) | 24823 (6315) | 16040 (1877) | 12145 (993) | 19301 (3080) | 22920 (3536) | 13219 (859) | 24725 (3350) |
| Gastro Intestinal | 12872 (916) | 18548 (619) | 20572 (1363) | 17006 (575) | 18016 (763) | 14363 (531) | 14058 (649) | 18509 (726) | 27956 (2224) | 13238 (439) | 15972 (672) | 27156 (1432) | 15645 (467) | 23389 (1082) |
| Neurological | 14402 (1259) | 19206 (1068) | 20855 (2056) | 21941 (1394) | 13676 (802) | 14212 (1124) | 14135 (1191) | 22736 (1865) | 31963 (3927) | 14236 (979) | 15722 (1371) | 27843 (1924) | 16478 (978) | 22300 (1427) |
| Musculoskeletal | 25043 (2743) | 21777 (1180) | 24352 (2071) | 24015 (1443) | 21554 (1392) | 14213 (976) | 25283 (2038) | 28271 (2686) | 35360 (3318) | 15820 (976) | 20399 (1791) | 30454 (2004) | 18228 (820) | 32387 (2230) |
| Genito Urinary | 15863 (1446) | 22429 (817) | 32546 (2435) | 25993 (1522) | 20937 (733) | 18960 (1010) | 20057 (1134) | 22023 (1246) | 37333 (3124) | 16031 (699) | 19067 (720) | 34771 (1917) | 22105 (858) | 27921 (1479) |
| Injuries | 16202 (2163) | 25085 (887) | 32461 (1928) | 26227 (988) | 21090 (1099) | 19809 (1022) | 22117 (1557) | 28620 (1682) | 29963 (1746) | 18464 (853) | 20408 (853) | 38959 (2068) | 22474 (845) | 30531 (1474) |
| Heart Diseases | 34241 (5860) | 29380 (1352) | 52876 (2538) | 45002 (1927) | 27797 (1613) | 21922 (1347) | 38804 (2613) | 46776 (3270) | 66323 (4260) | 21180 (1406) | 25263 (1322) | 63729 (2943) | 34589 (1776) | 49529 (2212) |
| Cancer | 47901 (11408) | 65070 (5696) | 45624 (4837) | 61935 (9831) | 52029 (5846) | 44154 (4227) | 61359 (11490) | 32414 (3407) | 93083 (19688) | 45538 (3735) | 50033 (7180) | 70190 (6893) | 56305 (5908) | 58712 (4755) |
| **All Diseases** | **12302 (328)** | **18915 (304)** | **24640 (602)** | **20372 (341)** | **15477 (260)** | **13502 (230)** | **16788 (421)** | **20309 (465)** | **28449 (857)** | **12391 (234)** | **15777 (297)** | **30370 (618)** | **16558 (269)** | **24107 (433)** |
| Communicable Diseases | 9077 (230) | 11086 (269) | 11718 (607) | 11207 (313) | 9724 (223) | 9471 (255) | 9230 (321) | 11088 (453) | 15183 (744) | 7784 (182) | 9598 (248) | 16180 (557) | 9236 (183) | 13456 (393) |
| NCDs | 21599 (1518) | 25523 (678) | 34912 (1142) | 31233 (894) | 21613 (637) | 18259 (564) | 27032 (1162) | 27981 (1032) | 44013 (2228) | 17690 (553) | 21995 (789) | 41976 (1237) | 25182 (742) | 33892 (903) |
| Injuries | 16202 (2163) | 25085 (887) | 32461 (1928) | 26227 (988) | 21090 (1099) | 19809 (1022) | 22117 (1557) | 28620 (1682) | 29963 (1748) | 18464 (853) | 20408 (853) | 38959 (2068) | 22474 (845) | 30531 (1474) |

^#^ 1 USD= 60.745 INR at 2014 exchange rates.
